# Selective digestive decontamination, a seemingly effective regimen with individual benefit or a flawed concept with population harm?

**DOI:** 10.1186/s13054-021-03744-w

**Published:** 2021-09-01

**Authors:** James C. Hurley

**Affiliations:** 1 Melbourne Medical School, University of Melbourne, Melbourne, Australia; 2 Division of Internal Medicine, Ballarat Health Services, PO Box 577, Ballarat, VIC 3353 Australia

**Keywords:** Bacteremia, Mechanical ventilation, Selective digestive decontamination, Polymyxin

## Abstract

Selective digestive decontamination (SDD) regimens, variously constituted with topical antibiotic prophylaxis (TAP) and protocolized parenteral antibiotic prophylaxis (PPAP), appear highly effective for preventing ICU-acquired infections but only within randomized concurrent control trials (RCCT’s). Confusingly, SDD is also a concept which, if true, implies population benefit. The SDD concept can finally be reified  in humans using the broad accumulated evidence base, including studies of TAP and PPAP that used non-concurrent controls (NCC), as a natural experiment. However, this test implicates overall population harm with higher event rates associated with SDD use within the ICU context.

## Introduction

Selective digestive decontamination (SDD) is both a variously constituted antibiotic regimen and an unreified concept. SDD regimens appear highly effective for preventing ICU-acquired infections [1–4]. The SDD concept arose 50 years ago within experiments to improve supportive care for neutropenic patients. Like vaccination interventions, the SDD concept has infection prevention implications for both individual patients and populations [5–8].

**Fig. 1:**
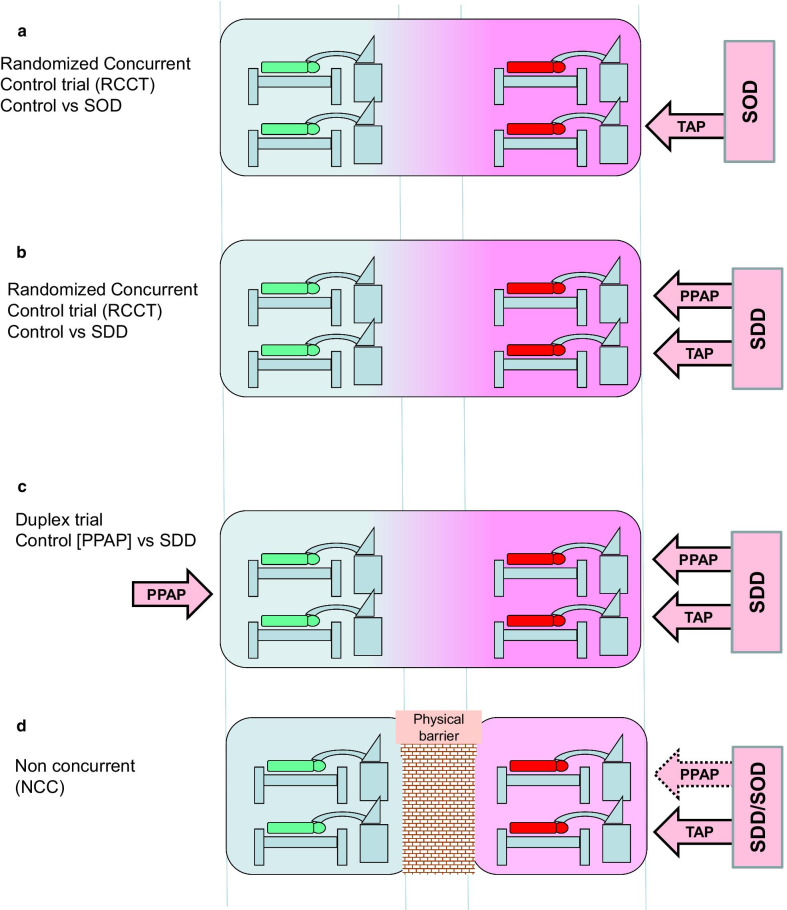
Schematic of ICU patients as intervention (right) and control (left) groups receiving mechanical ventilation in an ICU. The intervention and control groups are either concurrent (**a**–**c**), or non-concurrent (**d**) and separated by a physical or temporal barrier. The intervention groups receive topical antibiotic prophylaxis (TAP) with (**b**,** c**,** d**) or without protocolized parenteral antibiotic prophylaxis (PPAP) (**a**, **d**). Duplex studies are those where control group patients received PPAP (RCCT-duplex studies; **c**). The colour gradient indicates that the contextual effect emanating from intervention patients within the ICU that is implied by the SDD concept associated with the use of TAP

SDD has multiple confusing aspects. The applications and composition of SDD regimens have drifted far from that originally conceived. It is neither a single regimen nor protocol. The mode of action, benefits and associated risks remain unclear despite extensive study among various ICU, haematology and other immunocompromised patient groups. The SDD studies used different study designs (Fig. 1), different end points and different target populations.

This breadth of study findings, within studies of regimens using one or both of the two main SDD components, ‘TAP’ and ‘PPAP’, provides a unique and valuable natural experiment with which to test the SDD concept and its implications to the ‘whole of ICU’ population.

Here, SDD is referred to by its two main components ‘TAP’ and ‘PPAP’, so as to minimize ambiguity between SDD regimens and the unreified SDD concept.

### SDD, the conceptual origin

The SDD concept arose fifty years ago when neutropenia was the major limiting factor towards developing effective anti-leukemic chemotherapy. Pseudomonas and other Gram-negative (GN) bacteremias complicating chemotherapy-associated neutropenia were associated with high mortality. In this era, with few effective anti-pseudomonal antibiotics, preventing acquired infections among high-risk neutropenic patients required protective isolation [8, 9].

The SDD concept arose from studies using irradiated chimeric mice transplanted with leukemic cells which were at high risk of lethal blood stream infection arising from the digestive tract [5–7]. Earlier experiments demonstrated that increased susceptibility to oral challenge with *Salmonella enteritidis* was associated with loss of normally resident intestinal flora following prior antibiotic exposure. The novel observation within neutropenic mice was that this susceptibility varied depending on the timing and scale of the experimental antibiotic and irradiation exposures and that ‘selective’ antibiotic exposure prevented  lethal bacteremia following radiation. These experimental mice were at high risk without protective isolation in germ-free conditions. A cross-infection incident with resistant Gram-negative bacteremia, arising from monkeys housed in the same research facility, dramatically illustrated this susceptibility with extensive loss of experimental mice [7].

Five surprising observations emerging from these seminal experiments underpin the SDD concept [5–8]. Firstly, exposure to orally administered antibiotics sparing the anaerobic flora enabled the mice to tolerate radiation doses two Gy higher versus mice not antibiotic exposed. Second, oral administration of single antibiotics, such as streptomycin, kanamycin or neomycin, resulted in better survival and lower incidences of bacteremia versus exposure to all three antibiotics in combination. Presumably, single antibiotic exposure caused ‘selective effects’ on the intestinal flora. Third, the risk associated with loss of colonization resistance was quantifiable as the oral bacterial dose required to establish intestinal tract colonization in 50% of mice following challenge with bacteria such as *Klebsiella pneumoniae* or *Pseudomonas aeruginosa*. For example, mice lacking colonization resistance sustained colonization levels that were 10^5^-fold higher and required lower challenge doses, being 10^6^-fold lower. The colonization resistance time course was characteristic, a nadir at four days with recovery at three weeks following antibiotic exposure. The recovery corresponded to the time taken to clear challenge bacteria from the intestinal tract. Finally, most surprisingly, on recovery, this colonization resistance was enhanced. Moreover, it was transferable as a contextual effect. That is, germ-free mice acquired colonization resistance  from recovered mice housed in the same cage, or even simply being housed in cages contaminated by faecal flora from recovered mice [5].

With the SDD concept seemingly unverifiable in humans, as replicating the challenge studies undertaken in irradiated chimeric mice was not possible, translation of the concepts into clinical applications followed. As colonization resistance appeared most closely associated with anaerobic flora, a ‘traffic light’ classification of therapeutic antibiotics for at risk patients resulted. ‘Red light’ antibiotics, such as amoxicillin and clindamycin, with known activity against anaerobic flora, were to be avoided to minimize loss of colonization resistance [8].

The results of limited human volunteer colonization resistance experiments are difficult to summarize. It was highly variable between human volunteers and difficult to quantify. Notably, some findings in humans differed to those in experimental mice. The traffic light classification of antibiotics was abandoned and two ‘amber light’ antibiotics, trimethoprim–sulfamethoxazole and cefotaxime, each having potential anti-anaerobic activity at higher doses, were later adopted as key components of SDD regimens used among haematological and mechanically ventilated ICU patients, respectively [8, 10].

### SDD regimen change

Ideally, the properties of enteral (TAP) antibiotics would include activity against Pseudomonas, being non-absorbable (to maximize activity within the bowel lumen), well tolerated and cheap. As no one antibiotic agent satisfied all criteria, more than 20 TAP regimens with various combinations of two or three enteral antibiotics, such as polymyxin, tobramycin, gentamicin, netilmicin or nalidixic acid, were empirically derived.

Confusingly, other ‘SDD’ regimens developed to prevent bacteremia in neutropenic patients included agents systemically adsorbed after oral administration, such as trimethoprim–sulfamethoxazole, ciprofloxacin, norfloxacin, ofloxacin or cefuroxime. Whether these ‘SDD’ regimens mediated the prevention effect via intestinal decontamination versus systemic effects following adsorption is unclear.

Usually, SDD regimens include an anti-fungal agent to minimize yeast overgrowth. Surprisingly, among 33 RCCT’s among critically ill patients, SDD regimens outperformed single-agent anti-fungal prophylaxis with respect to reducing yeast colonization, invasive yeast infection and in-hospital mortality [11].

The duration and frequency of SDD regimen applications generally corresponds to time at risk being the duration of either neutropenia or mechanical ventilation. Regimens developed for ICU patients additionally include PPAP, such as such as cefotaxime, ceftriaxone and ceftazidime, for the first four days. Whether PPAP provides ‘pre-emptive therapy’ for infections potentially incubating on admission or provide interim coverage while the TAP components decontaminate the entire intestinal tract, is unclear. The exclusion from analysis of patients within SDD trials who are either extubated or died within the initial four days potentially creates immortal time bias.

Confusingly, regimens without PPAP, such as TAP applications to the oropharynx, being selective oropharyngeal decontamination (SOD), developed specifically for pneumonia prevention, unexpectedly also prevented bacteremia [10]. Also, within some RCCT’s, concurrent control group patients received PPAP (duplex studies, [Fig. 1c]) to mitigate cross-infection risks. Of note, the summary VAP and mortality prevention effects derived from duplex RCCT’s are not significant [1].

Trauma patients, whose colonization resistance is likely intact on admission to ICU, are considered ideal for SDD [12]. While early analyses indicated better mortality outcomes for surgical or trauma versus medical patient subgroups, more recent analyses showed similar apparent benefits among ICU patient subgroups [2, 13, 14].

### SDD a triple misnomer

Given the above, it is unclear whether SDD regimens mediate effects through selective removal (decontamination) of pathogens from the gut colonizing flora, whether the optimal location of decontamination is the gut or elsewhere, what the respective roles of TAP versus PPAP are towards the mediation of SDD effect, and whether complete decontamination is required. Some have studied whether polymyxin prevents adsorption of endotoxin from the bowel lumen as being an explanation for SDD effectiveness among ICU patients. In light of these uncertainties, the term ‘SDD’ is a triple misnomer and ‘control of gut overgrowth (COGO)’ better describes the presumed mediation [15, 16].

### SDD in neutropenia

In the 1970s, it was hoped that SDD regimens (nearly always TAP) used post-chemotherapy would enable neutropenic patient care without protective isolation [8, 9, 17, 18]. Clinical trials comparing protective isolation versus TAP used separately and together in neutropenic patients are confusing and their relative merits were unclear [17]. While the combination appeared to be beneficial, the studies were heterogeneous, were often small (< 50 patients per group), random allocation depended on the isolation unit availability at the time of random assignment, and protective isolation was difficult to standardize and moreover, psychologically distressing. Also, the SDD regimens varied and were poorly tolerated. For example, the taste of TAP using oral gentamicin, nystatin and vancomycin (GVN) was described as ‘dreadful’. Of great concern, the premature discontinuation of GVN resulted in rebound recolonization by Pseudomonas and Candida species in the gut causing bacteremia and other infections [9, 17].

Despite numerous SDD studies among haematological patients, many questions, such as optimal component antibiotics, optimal end points, mechanism of action and population effects, remained unanswered. The results of SDD studies among haematological and other patient groups failed to demonstrate the apparent success that later emerged from SDD studies among ICU patients receiving mechanical ventilation [1].

After 1980, interest in SDD use among neutropenic patients waned for three reasons. Effective anti-pseudomonal coverage for febrile neutropenia episodes became available. Well-tolerated single-agent prophylaxis regimens, using either trimethoprim–sulfamethoxazole or the newly available quinolone agents, had been demonstrated to reduce morbidity and numbers of Gram-negative infections in neutropenic patients. Rebound among high-risk patients after premature TAP withdrawal lingered as a safety concern. Recent European guidelines and commentaries on neutropenia infection prevention do not mention SDD regimens [18].

### Pivot to ICU patients

Patients receiving more than 48 h of mechanical ventilation frequently develop ventilator-associated pneumonia (VAP) with Pseudomonas, and other GN bacteria, acquired during hospitalization. VAP and other acquired infections increase mortality and are difficult to treat.

The summary evidence from sixteen early RCCT’s (3361 ICU patients) implied that only five and 23 patients would need to be treated with SDD to prevent one infection and one death, respectively [19]. This evidence, recently updated with results from 41 RCCT’s (11,004 ICU patients) [1], enables TAP to be compared versus five other VAP prevention interventions (Table 1) [20–23] using summary data as tabulated within recent Cochrane reviews. TAP appears highly effective towards preventing VAP versus these other interventions and, as TAP combined with PPAP, appears to be the only intervention effective at reducing mortality.

**Table 1:** Summary of findings from Cochrane reviews of VAP prevention interventions ^a^

Intervention	Ref	VAP incidence (per 1000 patients)				Mortality incidence (per 1000 patients)			
		Control	Intervention	RR; (95% CI)	n/N	Control	Intervention	RR; (95% CI)	n/N
Semi-recumbent ^b^	[20]	316	139	0·44; 0·11–1·77	3/419	276	240	0·87; 0·59–1·27	2/307
HME ^c^	[21]	167	155	0·93; 0·73–1·19	13/2251	247	257	1·03; 0·89–1·2	12/1951
Probiotic ^d^	[22]	309	238	0·7; 0·52–0·95	8/1018	214	186	0·84; 0·58–1·22	5/703
Chlorhexidine ^e^	[23]	243	180	0·75; 0·62–0·91	18/2451	222	242	1·09; 0·96–1·23	14/2014
Tooth brushing ^f^	[23]	253	206	0·69; 0·44–1·09	5/889	269	237	0·87; 0·7–1·09	5/889
TAP + PPAP ^g^	[1]	417	179	0·43; 0·35–0·53	17/2951	303	255	0·84; 0·73–0·96	18/5290
TAP (alone) ^h^	[1]	324	162	0·50; 0·36–0·69	13/1848	305	296	0·97; 0·87–1·07	15/3274
TAP + PPAP (versus PPAP) ^i, j^	[1]	303	278	0·82; 0·58–1·16	6/247	237	221	0·92; 0·72–1·18	7/1039

^a^n/N is number of participants/number of studies

^b^Semi-recumbent position; pneumonia is microbiologically confirmed VAP at > 48 h and mortality is ICU mortality at > 48 h

^c^HME (heat and moisture exchanger); pneumonia measured at a median 4 days (from Analysis 1.3 on page 65 of ref [21]) and mortality measured at a median 8 days

^d^Probiotic; pneumonia is VAP measured at a median 37 days and mortality measured at a median 35 days

^e^Chlorhexidine (mouth rinse or gel); pneumonia is VAP measured at a median 1 month and mortality measured at a median 1 month

^f^Toothbrushing; pneumonia is VAP measured at a mean 1 month and mortality measured at a mean 1 month

^g^TAP + PPAP studies; pneumonia is respiratory tract infection at unspecified follow up and mortality is at unspecified follow up. These studies resemble those having designs as in Fig. 1b

^h^TAP alone; pneumonia is respiratory tract infection at unspecified follow up (note this does not include 6 studies for which the control group received PPAP) and mortality is at unspecified follow up. These studies resemble those having designs as in Fig. 1a

^i^TAP + PPAP versus PPAP (duplex studies); pneumonia is respiratory tract infection at unspecified follow up and mortality is at unspecified follow up. These studies resemble those having designs as in Fig. 1c

^j^Note that only one study [10] having a NCC design (as in Fig. 1d) is included within the systematic review of TAP[1]

**Fig. 2:**
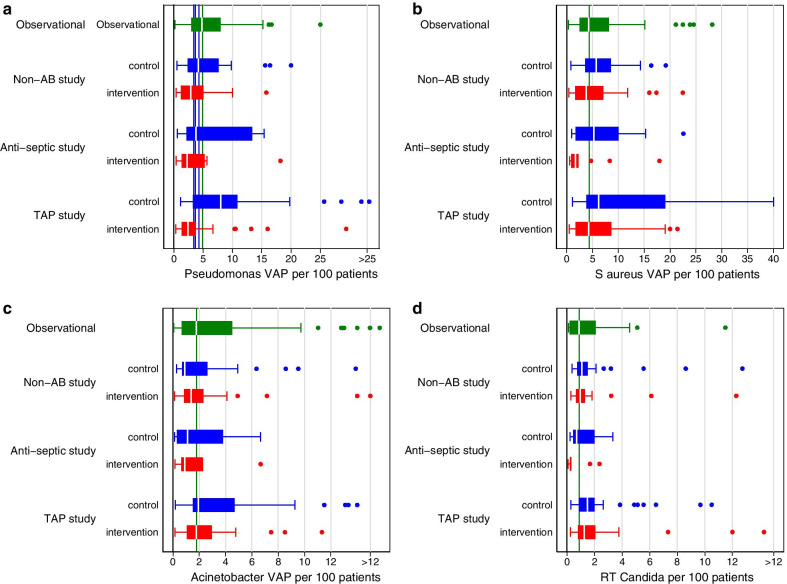
Boxplots displaying the median (centre line), IQR (outer limits of box), upper and lower limits (whiskers) and outlier observations (dots) for VAP incidence from groups with > 50% receiving MV where the identified microbe is *Pseudomonas* (**a**), *Staphylococcus aureus* (**b**), *Acinetobacter* (**c**) or *Candida* species (**d**) [data from between 113 and 191 studies (catalogued in [24–27]). The overall benchmark (vertical green line) is the median derived from observational groups (green). Non-AB study is studies of non-antibiotic-based methods of infection prevention; TAP is topical antibiotic prophylaxis. Of note, the benchmark incidence for Pseudomonas VAP here (4.7 per 100 patients) is similar to that in a multi-centre survey [28]. The vertical blue lines are the Pseudomonas VAP incidence observed in ICU’s in Europe (4.8%), the USA (3.4%) and the Asia–pacific regions (3.2%) in the multi-centre survey [28]

Comparing the counts of VAP with various specific bacteria isolated (Fig. 2) reinforces the impression that, in contrast to five other interventions, in two broad categories drawn from the evidence base (Table 1), SDD has profound and selective anti-pseudomonal effects [24–28].

**Fig. 3:**
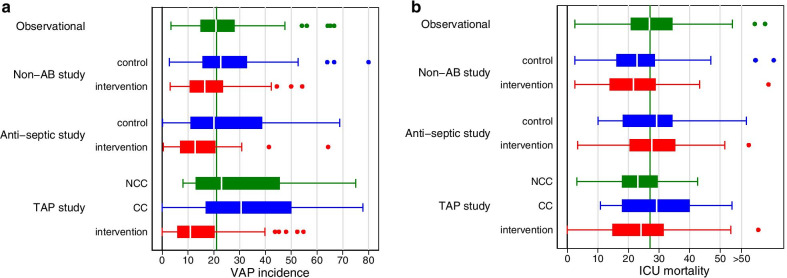
Boxplots displaying the median (centre line), IQR (outer limits of box), upper and lower limits (whiskers) and outlier observations (dots) for VAP incidence (**a**) and ICU mortality (**b**) from studies where this was available (bottom) from concurrent control (blue), and NCC control (green) and intervention (red) groups with > 50% receiving MV [data from 206 (studies catalogued in [32]). The overall benchmark (vertical green line) is the median derived from observational groups (green). CC is concurrent control (coming from RCCT design studies as in Fig. 1a–c), or non-concurrent (NCC as in Fig. 1d); non-AB study is studies of non-antibiotic-based methods of infection prevention; TAP is topical antibiotic prophylaxis (with or without PPAP)

However, several puzzling observations emerge from this literature reappraisal. Surprisingly, the median VAP and mortality incidences among the TAP intervention groups are similar to (i.e. not lower than) the other intervention group medians (Table 1, Fig. 3).

Likewise, the median Pseudomonas VAP incidence is similar versus the two broad intervention categories (Fig. 2a) and versus benchmark incidences derived either from the literature or global surveys [28]. Curiously, for VAP where either Staphylococcus, Acinetobacter or Candida have been isolated, the majority of control groups of TAP studies and at least half of TAP intervention groups have incidences above the respective benchmarks (Fig. 2) [24–28].

Comparing RCCT group incidences, unadjusted for underlying patient risk, from disparate studies, unweighted for study size or quality (Table 1 and the boxplots as in Fig. 2 & 3), could be criticized. Such comparisons of group level incidences across studies of diverse design, patient mix and origin are ecological, simplistic and poorly reflect patient level TAP effects. Such comparisons, using group level incidences in the estimation of causal effects of interventions for individual patients, are widely discredited. Also, VAP diagnosis is potentially subjective, and its microbiological documentation potentially masked by TAP and anti-septic interventions.

While simplistic, these broad comparisons across the evidence base enable an appraisal of TAP population effects within the ‘whole of ICU’. Of note, in contrast to estimating individual patient level effects of interventions, determining the population effect of any intervention is not possible within any one RCCT or even within several RCCT’s of a single intervention.

That TAP effects would extend beyond individual recipients, mediated by cross-infection, as had been noted in the original colonization resistance experiments in mice, created an expectation of benefit to concurrent non-recipients within RCCT’s of TAP [12]. Cross-infection, whether aerobic flora from control group to intervention group patients or, anaerobic flora from intervention group to control group patients, within conventional RCCT’s would undermine estimates of TAP efficacy. Cross-infection of either type, being inapparent within even moderate sized RCCT’s [29], would bias RCCT results towards the null [12]. This expectation, based on testable postulates [24], prompted further evaluation within studies using NCC rather than RCCT design.

### Pivot to ICU non-concurrent controlled trials

NCC studies mitigate these cross-infection threats by segregating enrolled patients, either into separate ICU’s or, non-concurrent periods in the same ICU. The NCC design (Fig. 1d), where the unit of randomization is the ICU, not the patient, also has logistical benefits. However, substantially more patients are required to adequately power CRT’s due to the non-independence of observations within each ICU [30]. Also, blinding within NCC design studies is difficult and bacteremia, being less subjective than VAP, is the preferred end point.

Despite expectations, the results of three large [each > 5000 patients and > 13 ICU’s] CRT’s were marginal compared to RCCT’s results (Table 1) [10, 13, 31]. A fourth is in progress. One found similar overall mortality within ICU’s randomized to SOD, SDD or standard care with significant differences emerging after adjusting for differences in underlying patient mortality risk [10]. The second found lower day-28 mortality and ICU-acquired bacteremia associated with SDD versus SOD but lacked standard care control groups [13]. The third, and largest, found no reduction, with either SDD or SOD, in either bacteremia or mortality even in adjusted analyses [31].

Of concern, the statistical adjustment underlying the significant results obtained in the first CRT [10], and in subsequent individual patient data meta-analyses [2], fails to account for any carry-over of contextual ‘whole-of-ICU’ effects between TAP and control periods.

Moreover, most surprisingly, the median incidence for VAP, bacteremia and ICU mortality among NCC control groups are each closer to literature benchmarks versus RCCT control groups (Fig. 3) [24–28]. These paradoxical observations, arising also in comparisons of blood stream infections, whether overall or for specific types, and ICU mortality end points, are each unexplained in regression models and invite a reappraisal of other ‘whole of ICU’ consequences of TAP exposure [32–37].

### Rebound

Patient outcomes following SDD cessation and following ICU discharge are an area of increasing research. Hospital-acquired infections are up to 50% more common among patients discharged after receiving either SDD or SOD during ICU stay versus patients discharged following standard care [38].

Rebound follows cessation of TAP [39–45]. Rebound following premature cessation of TAP in haematology patients was a major concern [9]. *P. aeruginosa* rebound among ICU patients occurred following both continuous TAP use [41] and aerosolized polymyxin use as prevention during alternate months [42]. The optimal time for washout after SDD/SOD withdrawal to avoid rebound carrying-over into subsequent CRT control periods is unclear.

Resistant GN microorganisms, colonizing respiratory and rectal sites, rebound following periods of TAP use to levels higher than in baseline periods. This rebound occurs among the ‘whole of ICU’ population [39]. Whether antibiotic-sensitive bacteria likewise rebound is unclear and difficult to study except in mice. Patients receiving TAP can serve as reservoirs for *Pseudomonas* strains appearing among control group infections [40]. Whether rebound occurs within the intestinal ‘resistome’, which includes non-culturable flora, and the broader question of consequences for antibiotic resistance and even for bacteria which exhibit antibiotic dependency, are areas of active investigation [46, 47]. Likewise, Candida interacting with bacteria within the microbiome might promote invasive bacterial infection from higher Candida colonization resulting from TAP [49].

### Herd effects of SDD

Herd effects, of great consequence to non-recipient individuals concurrent within populations exposed to vaccine interventions, cannot be estimated within single populations examined in isolation. These estimates require thousands of participants within multiple exposed and unexposed neighbourhoods to achieve adequate study power. For example, a CRT demonstrating typhoid vaccination herd effects enrolled 60,000 residents across 40 contiguous Eastern Kolkata neighbourhoods [50].

Estimating TAP herd effects, a crucial ‘whole of ICU’ question, would face multiple challenges. Three small studies (each < 200 patients and < 3 ICU’s) were inconclusive. The two largest (> 5000 patients and 13–16 ICU’s [10, 31]) CRT’s of TAP completed to date were underpowered to detect any direct TAP effect on any mortality end point in unadjusted analyses. Additionally, TAP herd effects would be diffused across multiple microbial end points, unlike typhoid vaccination studies wherein typhoid fever is the only relevant end point.

Finally, the greatest challenge in studying TAP herd effects is the participation of non-recipient patients. In consenting them for participation, is there equipoise regarding whether non-recipients indirectly derive benefit versus harm from being concurrent to TAP recipients in the ICU? What evidence underlies this equipoise? For vaccine interventions, herd protection with benefit to non-recipients appears plausible from the accumulated history of immunity resulting from outbreaks and vaccine interventions. What of the accumulated history with TAP use within ICU’s?

**Fig. 4:**
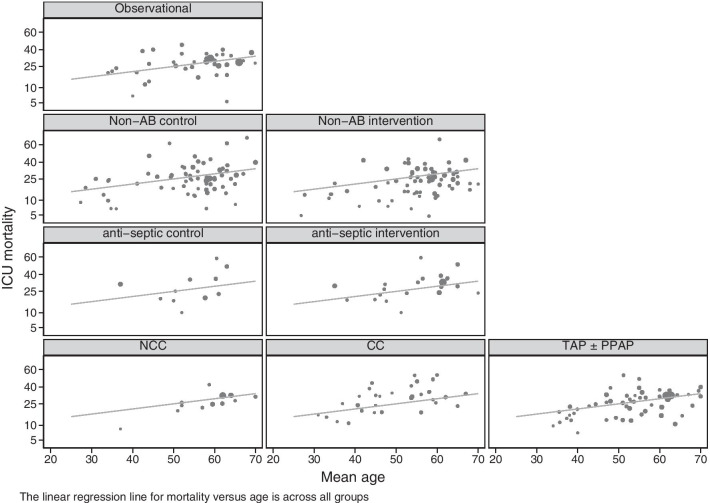
ICU mortality incidence among 175 groups from studies of observational (top row), and control (left most and middle panels) and intervention (right most panels) groups of non-antibiotic (second row), topical anti-septic (third row) and topical antibiotic (bottom row) studies of VAP prevention versus group mean age. Also shown for reference is the linear regression line of ICU mortality incidence versus group mean age as derived across all groups. The figure is adapted from reference [32] and used here under the terms of the Creative Commons Attribution 4.0 International License (http://creativecommons.org/licenses/by/4.0/)

More simply, ‘flipping’ the entire evidence base of VAP prevention interventions to simulate a multi-centre CRT of TAP use among ICU’s enables a reappraisal of the ‘whole of ICU’ effects of TAP. This ‘natural experiment’ [32–37] identifies control groups that are either exposed versus not exposed to concurrent patients receiving TAP within the ICU. The higher incidences of ICU mortality [32], which remains evident within a regression adjusted for group mean age (Fig. 4), and several infections among exposed non-recipients (i.e. concurrent control group patients within TAP RCCT’s) versus those not exposed (Fig. 3) are unexplained. On the other hand, the similarity of the incidence of these various end points among TAP intervention groups with benchmarks and with the incidences among all other intervention and control groups of various types, with the notable exception of RCCT control groups (Table 1), raises doubt about the population safety of TAP.

## Conclusion

Strangely, despite high apparent efficacy of SDD regimens, within ICU based RCCT’s, the concurrent control group incidences of several infections and mortality are higher versus other groups including groups within NCC studies of SDD regimens. These results are contrary to the original SDD concept. Surprisingly, the incidences within the TAP intervention groups are closer to literature-derived benchmarks. While these discrepancies remain unexplained, SDD regimens appear unsafe for use in the ICU.

## Data Availability

Supportive data are available in the author’s cited publications [16, 24–27, 29, 32–37, 49].

## Abbreviations

COGO: Control of gut overgrowth
GN: Gram-negative
ICU: Intensive care unit
MV: Mechanical ventilation
PPAP: Protocolized parenteral antibiotic prophylaxis
NCC: Non-concurrent control
CC: Concurrent control
RCCT: Randomized concurrent control trial
SOD/SDD: Selective digestive decontamination/selective digestive decontamination
TAP: Topical antibiotic prophylaxis
